# Expression of H3K4me3 and H3K9ac in breast cancer

**DOI:** 10.1007/s00432-020-03265-z

**Published:** 2020-05-28

**Authors:** Luisa Berger, Thomas Kolben, Sarah Meister, Theresa M. Kolben, Elisa Schmoeckel, Doris Mayr, Sven Mahner, Udo Jeschke, Nina Ditsch, Susanne Beyer

**Affiliations:** 1 Department of Obstetrics and Gynecology, University Hospital, LMU Munich, Marchioninistr. 15, 81377 Munich, Germany; 2 Institute of Pathology, University Hospital, LMU Munich, Marchioninistr. 15, 81377 Munich, Germany; 3 Department of Obstetrics and Gynecology, University Hospital, Universitätsklinikum Augsburg, Stenglinstr. 2, 86156 Augsburg, Germany

**Keywords:** Histone modification, Breast cancer, Epigenetic, survival

## Abstract

**Purpose:**

Breast cancer is the leading cause of cancer death in females. Histone modifications have been shown to have an influence on the gene expression. This study focusses on the histone modifications H3K9ac and H3K4me3 in breast cancer and their impact on survival

**Methods:**

H3K4me3 and H3K9ac expression was immunohistochemically examined in 235 tissue samples.

**Results:**

Positive estrogen receptor status was correlated with a higher IRS of the nuclear (*p* = 0.033), and of the cytoplasmic H3K4me3 staining (*p* = 0.009). H3K9ac intensity was associated to the Her2 status (*p* = 0.045) and to poor prognosis in cells with positive Ki67 status (*p* = 0.013). A high intensity of nuclear H3K4me3 staining was found to be correlated with a lower 10-year-survival (*p* = 0.026) and with lower breast cancer-specific survival (*p* = 0.004). High percentage score (> 190) of H3K9ac expression was correlated to worse breast cancer-specific survival (*p* = 0.005). Shorter progression-free survival was found in patients with nuclear (*p* = 0.013) and cytoplasmic H3K4me3expression (*p* = 0.024) and H3K9ac expression (*p* = 0.023).

**Conclusion:**

This analysis provides new evidence of histone modifications in breast cancer. High H3K4me3 and H3K9ac expression was correlated with survival rates. Further investigation of histone modifications in breast cancer could lead to a more profound understanding of the molecular mechanisms of cancer development and could result in new therapeutic strategies.

## Introduction

Breast cancer is the most frequently diagnosed cancer and the leading cause of cancer death in females worldwide, accounting for 25% (1.7 million) of the total new cancer cases and 15% (521.900) of the total cancer-related deaths in 2012 (Torre et al. 2017). Breast cancer remains a significant threat to women all over the world, even though the breast cancer death rates have decreased by 40% between 1975 and 2017 (DeSantis et al. 2019).

Gene expression profiling has had an important impact on the understanding of breast cancer (Bell et al. 2017). For example, the analysis of certain biomarkers such as hormone receptor status, Her2 status and expression of Ki67 has led to the characterization of molecular subtypes of breast cancer that have shown significant differences in terms of their incidence, risk factors, prognosis and sensitivity to treatment (Prat et al. 2015).

Epigenetic alterations, such as DNA methylation and posttranslational modification of histones have been shown to have a considerable influence on the gene expression (Wu et al. 2015). Histones are the central component of the nucleosomes’ subunit. They form an octamer containing the four core histone proteins (H3, H4, H2A, H2B) around which is wrapped a 147-base-pair segment of the DNA (Audia and Campbell 2016). The histones’ N-terminal tails extend from the double-strand DNA and are subject to posttranslational modifications, which include acetylation, methylation, phosphorylation, ADP-ribosylation, glycosylation, sumoylation and ubiquitylation (Zhang et al. 2016). Histone acetylation is primarily associated with gene activation, whereas methylation, depending on its position and state, can either be associated with repression or activation (Wang et al. 2008).

Previous studies have analyzed the impact of specific posttranslational modifications on the gene expression (Lawrence et al. 2016). For example, H3K4 methylation has been intensely studied regarding the enzymes and molecular factors required for methylation (Shilatifard 2008). An association between high H3K4me3 expression and poor prognosis was found in patients with hepatocellular carcinoma and cervical carcinoma (Beyer et al. 2017; He et al. 2012). In cervical cancer, the same observation has been made for H3K9ac (Beyer et al. 2017).

As a thorough investigation regarding the influence of histone modifications on the prognosis of breast cancer patients was lacking, an expression analysis of histone H3 trimethyl K4 (H3K4me3) and histone H3 acetyl K9 (H3K9ac) was performed in this study. 235 tissue samples were examined by immunohistochemical methods and assessed by a semi-quantitative score.

## Materials and methods

### Patients and specimens

A panel of 235 tissue samples from patients who underwent surgery at the Ludwig-Maximilians-University, Munich between 1998 and 2000 due to malignant breast cancer was used. The mean patients’ age was 58.2 ± 13.3 years. 43.8% of the breast cancer cases were classified as Luminal A, 31.5% as Luminal B and Her2 negative, 6.8% as Luminal B and Her2 positive, 3.0% as Her2 positive and hormone receptor negative; 13.2% were triple negative carcinomas. 65.1% of the patients were diagnosed with a tumor size smaller than 2 cm, 28.1% had a tumor between 2 and 5 cm. At least one regional lymph node was affected in 39.6% of the cases. 7.2% were ranked as low grade, 36.6% as intermediate grade and 22.6% as high-grade tumors. In 33.6%, the grading-classification was missing. DCIS and LCIS fractions were observed in 51.1% of the cases. For more patients’ characteristics’ see Table 1.

**Table 1:** Patients’ characteristics

	*N*	%
Age (median)	58.2	–
Histopathology		
Luminal A	103	43.8
Luminal B		
Luminal B Her2 negative	74	31.5
Luminal B Her2 positive	16	6.8
Her2 positive, HR negative	7	3.0
Triple negative	31	13.2
NA’s	4	1.7
< 2 cm	153	65.1
2–5 cm	66	28.1
> 5 cm	1	0.4
T4	5	2.1
NA’s	10	4.3
Grade 1	17	7.2
Grade 2	86	36.6
Grade 3	53	22.6
NA’s	79	33.6
Lymph node		
N−	122	51.9
N+	93	39.6
NA’s	20	8.5
DCIS/LCIS		
DCIS/LCIS positive	120	51.1
DCIS/LCIS negative	107	45.5
NA’s	8	3.4
Progression (over 15.6 years)		
None	139	59.1
At least one	64	27.2
Not available	32	13.6
Survival (over 14.7 years)		
Right censured	149	63.4
Died	78	33.2
Not available	8	3.4

The endpoints were defined as following: OS = overall survival, period of time from the date of surgery until the date of death or date of last follow-up; DSS = disease-specific survival, period of time from the date of surgery until the breast cancer-dependent death; PFS = progression-free survival, period of time until local recurrence or metastasis were diagnosed; local PFS = period of time until a local recurrence was diagnosed; DDFS = distant disease-free survival, period of time until metastasis is diagnosed.

### Ethics approval

The tissue samples were originally collected for histopathological diagnostics. They were no longer used for clinical tests when being selected for this study. Patient data was anonymized and the authors were blinded for the patients’ information as well as for survival time during the analysis. The study was approved by the local ethics committee of the Ludwig-Maximilians-University of Munich (Reference No. 048-08; 2008) and was performed according to the Declaration of Helsinki.

### Immunohistochemistry

**Fig. 1:**
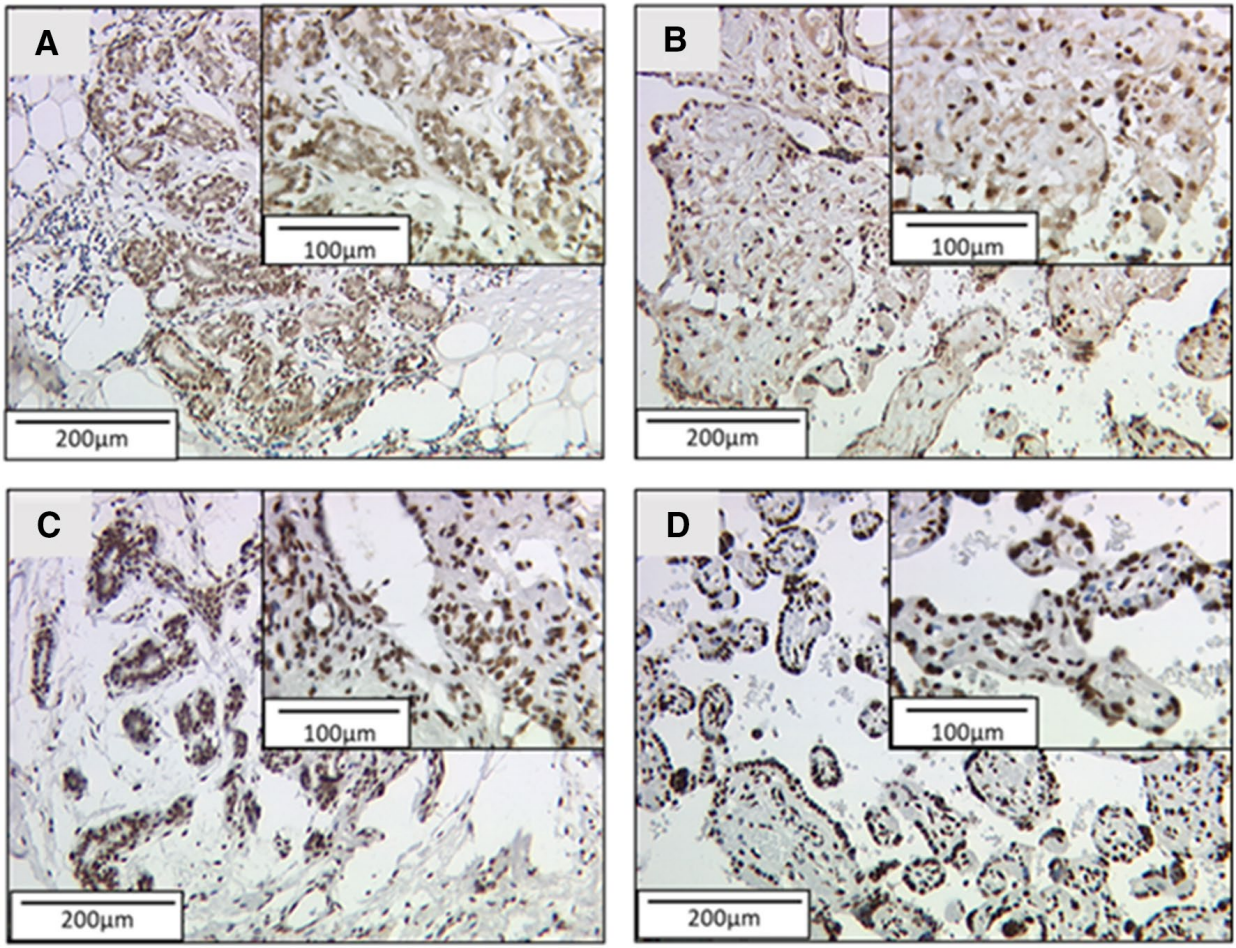
H3K4me3 and H3K9ac staining in BC and placenta. **a** H3K4me3 staining in BC; **b** positive control (placenta) H3K4me3; **c** H3K9ac staining in BC; **d** positive control (placenta) H3K9ac

The formalin-fixated and paraffin-embedded tissues were first dewaxed in xylol. After rinsing the tissue in 100% ethanol, the endogenous peroxidase was inactivated in 3% H_2_O_2_ in methanol and the samples were rehydrated in a descending alcohol series. To unmask the antigen, the samples were heated up to 100 °C in a dilution with citrate buffer for 5 min. After washing the samples in distilled water and PBS-buffer, a blocking solution was applied to prevent unspecific staining due to binding of the antibodies to electrostatic charges in the tissue. The samples were then incubated at 4 °C with the primary antibody for 16 h (see Table 2). A Purified solution of Antibodies bought from the producer was used. The specificity of the antibodies was already tested by CHiP Sequence by other authors and the producer (Abcam; Lima-Fernandes et al. 2019). After intensifying the staining with a Post-Block Solution, the HRP polymer was applied. The excess HRP Polymer was removed and the binding of the antibody was made visible through an enzymatic reaction using diaminobenzidine (DAB). A counterstaining was performed in haemalaun (2 min), followed by dehydration in an ascending alcohol series and covering of the samples. Placenta tissues were used as positive and negative controls for each staining of H3K4me3 and H3K9ac (Fig. 1b and d). The results of the staining were analyzed using two different scores. The immunoreactive score (IRS-Score) multiplies the intensity of the staining (0 = not stained, 1 = low intensity, 2 = moderate intensity, 3 = high intensity) with the percentage of stained tumor cells (0 = 0%, 1 = 1–10%, 2 = 11–50%, 3 = 51–80%, 4 =  > 80%). The result is a value between 0 and 12. The %Score multiplies the staining intensity (0 = not stained, 1 = low intensity, 2 = moderate intensity, 3 = high intensity) with the percentage of stained tumor cells, allowing a finer differentiation of the samples regarding the percentage of stained cells (range: 0–300%).

**Table 2:** Staining procedure

Histone H3 tri methyl K4^a^	Histone H3 acetyl K9^b^
Blocking solution^c^: 5 min	Blocking solution^c^: 5 min
Primary antibody^a^: 1:100 in PBS^d^, incubation: 16 h, 4 °C	Primary antibody^b^: 1:200 in PBS^d^, incubation: 16 h, 4 °C
PostBlock^c^: 20 min	PostBlock^c^: 20 min
HRP Polymer^c^: 30 min	HRP Polymer^c^: 30 min
Chromogen: DAB^e^ (1 min)	Chromogen DAB^e^ (30 s)

^a^Anti histone H3 tri methyl K4, rabbit IgG polyclonal, concentration: 0.2 mg/ml, company: Abcam, order number: ab8580

^b^Anti histone H3 acetyl K9, rabbit IgG monoclonal, clone Y28, concentration: 0.059 mg/ml, company: Abcam, order number: ab32129

^c^ZytoChem Plus HRP Polymer Kit (Mouse/Rabbit) 3 × 100, company: Zytomed Systems (Berlin, Germany) Nr. POLHRP-100

^d^Dulbecco’s phosphate buffered saline

^e^Lipuid DAB + substrate chromogen system 1 mg/ml, DAKO

### Statistics

IBM SPSS Statistics version 25 (Armonk, NY, USA) was used. Spearman’s-rank correlation coefficient was employed to calculate bivariate correlations. To compare independent groups non-parametric tests (NPAR: Kruskal–Wallis test, Mann–Whitney *U* test) were used. Survival times were shown by Kaplan–Meier estimates and calculated by log-rank-test, thresholds were defined experimentally. The *p* value of the tests had to be < 0.05 to be statistically significant.

## Results

### H3K4me3 staining in breast cancer

71.4% of the samples showed a nuclear staining with a median immunoreactive score of 2 and a median percentage score of 40 (Fig. 1a). 26.0% displayed no nuclear staining. 20.4% of the samples had low staining (IRS ≤ 2), while 44.3% showed an enhanced staining (IRS > 2) and, therefore, high expression of H3K4me3 in the nucleus. 9.3% of the samples could not be analyzed. An additional staining of the cytoplasm was found in 35.1% of the samples. Placenta tissue was used as positive control (Fig. 1b).

The correlations between the H3K4me3 expression and several important clinical parameters, such as the estrogen receptor status and the Her2 status, as well as the H3K9ac expression, were analyzed by applying Spearman’s rank correlation coefficient (see Table 3).

**Table 3:** Correlation of histopathological characteristics with the IRS staining

	H3K4me3 Nuc			H3K4me3 Cyt			H3K4ac		
	Median	*p*	*ρ*	Median	*p*	*ρ*	Median	*p*	*ρ*
	(± SD)			(± SD)			(± SD)		
Histology		0.808	0.017		**0.039**	**0.143**		0.309	0.069
DCIS/LCIS	2 (± 3.22)			0 (± 2.45)			3 (± 3.24)		
No DCIS/LCIS	2 (± 3.00)			0 (± 1.88)			3 (± 3.03)		
T-stage		0.738	0.023		0.086	− 0.12		0.109	− 0.109
< 2 cm	2 (± 3.22)			0 (± 2.43)			3 (± 3.29)		
2–5 cm	2 (± 2.67)			0 (± 1.62)			2 (± 2.67)		
> 5 cm	8 (± 0.00)			4 (± 0.00)			1 (± 0.00)		
T4	3 (± 2.08)			0 (± 2.23)			3 (± 3.14)		
pN		0.455	− 0.53		0.268	0.079		0.279	− 0.076
pN0	2,5 (± 3,14)			0 (± 2.05)			3 (± 3.30)		
pN1	2 (± 3,02)			0 (± 2.50)			3 (± 2.89)		
Grading		0.328	− 0.082		0.051	− 0.163		0.165	− 0.114
G1	3 (± 3.21)			1 (± 3.41)			3,5 (± 2.43)		
G2	3 (± 3.14)			0 (± 2.32)			3 (± 2.43)		
G3	2 (± 2.96)			0 (± 2.36)			3 (± 2.43)		
Ki67		0.835	− 0.017		0.443	0.061		0.704	− 0.029
Positive	3 (± 3.12)			0 (± 1.82)			3 (± 3.03)		
Negative	3 (± 3.08)			0 (± 2.33)			3 (± 3.23)		
ER		**0.033**	**0.147**		**0.009**	**0.179**		0.051	0.130
ER +	3 (± 3.12)			0 (± 2.20)			3 (± 3.17)		
ER-	2 (± 2.87)			0 (± 2.18)			2 (± 2.96)		
PR		0.164	0.096		0.512	0.045		0.411	0.055
PR +	3 (± 2.94)			0 (± 2.16)			3 (± 3.12)		
PR-	2 (± 3.32)			0 (± 2.23)			3 (± 3.21)		
Her2		0.761	0.021		0.109	0.11		**0.045**	**0.134**
Her2 +	2 (± 3.30)			0 (± 2.52)			4 (± 2.70)		
Her2-	2 (± 3.06)			2 (± 2.15)			3 (± 3.15)		
Clinical subtype	**–**	0.869	− 0.011	**–**	0.593	0.037	**–**	0.392	0.037
OS			**–**			**–**			**–**
Survival	2 (± 3.06)			0 (± 2.33)			3 (± 3.14)		
Death	3 (± 3.19)			0 (± 1.98)			3 (± 3.18)		
PFS			**–**			**–**			**–**
None	2 (± 3.10)			0 (± 2.09)			3 (± 2.96)		
Progression	3 (± 3.20)			0 (± 2.46)			3 (± 3.32)		

Significant results (*p* < 0.05) are shown in bold

*ρ* Rho

**Fig. 2:**
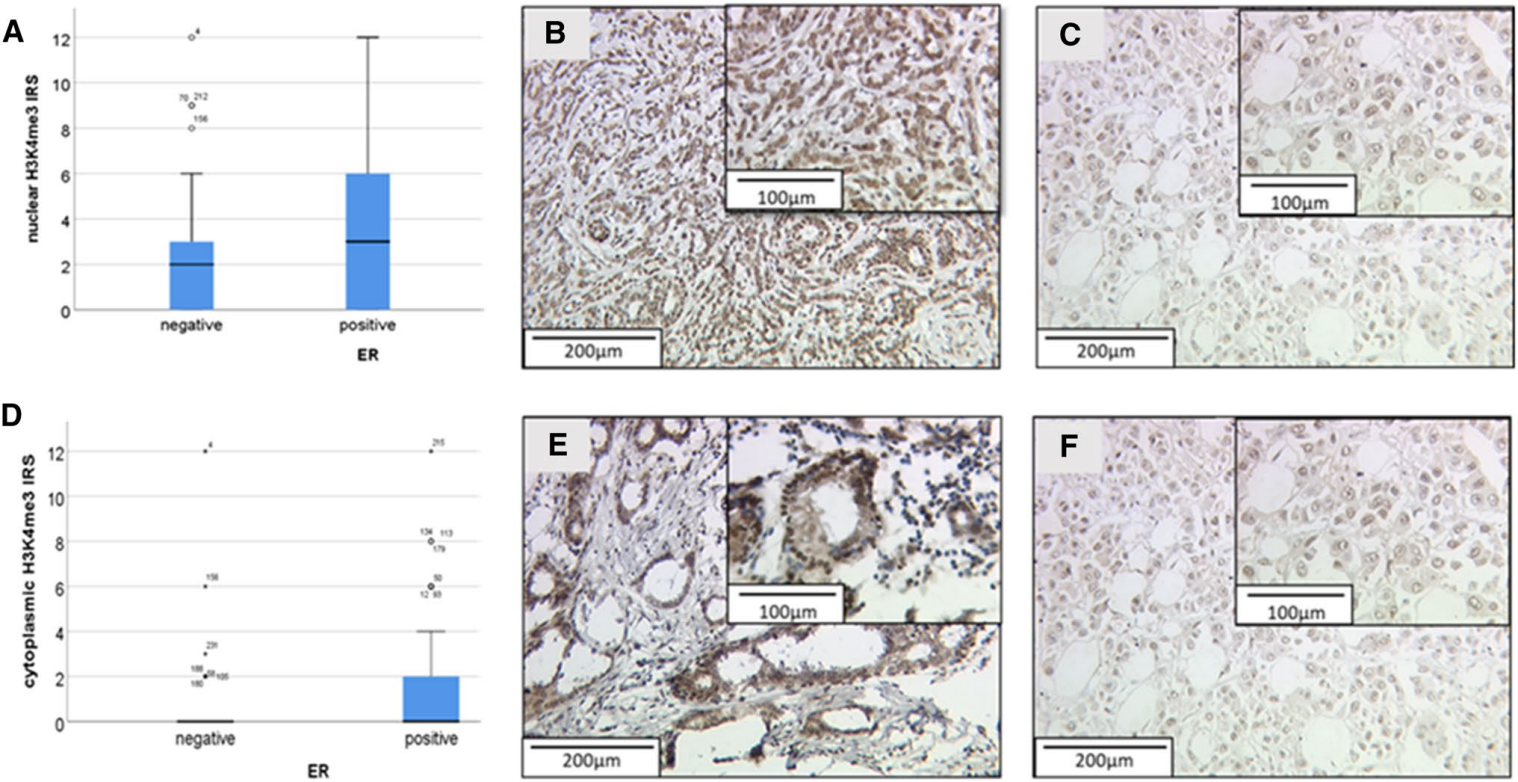
Correlation of H3K4me3 Nuc and Cyt and ER. **a** Boxplot: Difference of H3K4me3 Nuc expression in regard to ER status; **b** H3K4me3Nuc staining (IRS 9) and ER positive; **c** H3K4me3Nuc expression (IRS 2) and ER negative; **d** Boxplot: H3K4me3 Cyt expression in regard to ER status; **e** H3K4me3Cyt staining (IRS 8) and ER positive; **f** H3K4me3Cyt expression (IRS 3) and ER negative

An association between H3K4me3 and the tumor cells’ estrogen receptor status was observed: Positive estrogen receptor status was correlated with a higher IRS of the nuclear staining (p = 0.033, Rho = 0.147; Fig. 2a–c): the median of nuclear H3K4me3 expression in estrogen positive cells was 3, compared to 2 in estrogen negative cells. ER expression was also associated to a higher intensity (*p* = 0.006, Rho = 0.186) and IRS (*p* = 0.009, Rho = 0.179) of the cytoplasmic staining (Fig. 2d–f; see Table 3). Although the difference is significant, the correlation is weak and cannot be seen in the boxplot.

No significant correlation was found regarding the histological subtype, T-stage, N-stage, grading, Ki67 status, PR, Her2 status and clinical subtype (Luminal A, Luminal B, basal like triple negative Her2 positive typ luminal, Her2 positive typ non-luminal) (see Table 3).

A correlation between the H3K9ac staining and the nuclear H3K4me3 staining (*p* = 0.000; Rho = 0.633), as well as the cytoplasmic H3K4me3 staining (*p* = 0.000; Rho = 0.448) was found, showing that intense H3K4me3 staining is associated with increased H3K9ac expression (see Table 4).

**Table 4:** Correlation of H3K4me3 and H3K9ac

	H3K4me3 Nuc		H3K4me3 Cyt	
	*p*	*ρ*	*p*	*ρ*
H3K9ac	0.000	0.633	0.000	0.448

### H3K9ac staining in breast cancer

A total of 72.4% of the samples showed a nuclear staining with a median IRS of 3 and a median percentage score of 60 (Fig. 1c). Unlike the H3K4me3 staining, no additional staining of the cytoplasm was found. 28.5% of the samples had low staining (IRS ≤ 3), while 40.9% showed an enhanced staining (IRS > 3) and 26.4% did not show any staining. 4.2% of the samples could not be analyzed. Placenta tissue was used as positive control (Fig. 1d).

**Fig. 3:**
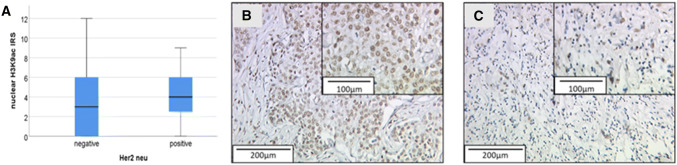
Correlation of H3K9ac and Her2neu. **a** Boxplot: correlation of Her2neuexpression and H3K9ac; **b** H3K9ac staining (IRS 8) and Her2neu positive; **c** H3K9acexpression (IRS 3) and Her2 negative

As mentioned above, a correlation between the H3K4me3 staining and the H3K9ac staining was found (see Table 4). Furthermore, a high H3K9ac staining intensity was shown to be correlated with a positive Her2 status of the tumor cells (*p* = 0.045, *ρ* = 0.134; Table 3; Fig. 3), but the correlation was weak.

**Fig. 4:**
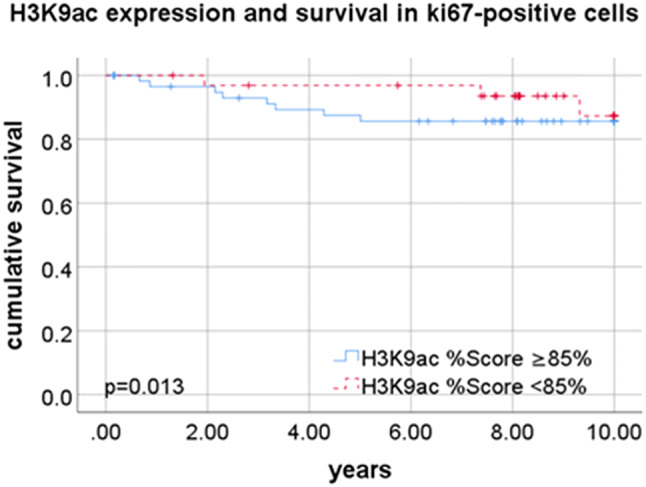
Correlation of H3K9ac and Ki67 and prognosis

The categorization of the molecular subtype is an important part of the breast cancer diagnosis, as it influences the patient’s treatment and allows to give an approximate prognosis. To further refine the prognosis in specific subcategories, more markers are useful. The expression of H3K9ac was identified as a potential marker. Regarding the Ki67 status, H3K9ac expression was found not to be directly associated to Ki67 expression. But in samples with positive Ki67 status (defined as more than 14% of the tumor cells being positive for Ki67), H3K9ac expression was associated with poor prognosis (*p* = 0.013; Fig. 4).

No significant correlation was found regarding the T-stage, N-stage, estrogen receptor status, grading, the PR and the clinical subtype.

### Role of H3K4me3 and H3K9ac on survival

**Fig. 5:**
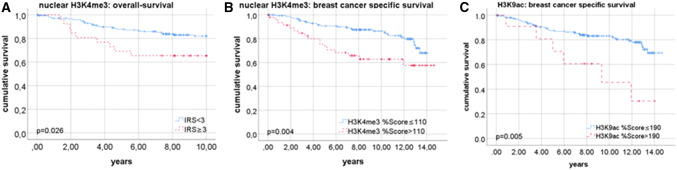
Role of H3K4me3 and H3K9ac for survival. **a** nuclear H3K4me3 expression and overall survival. **b** nuclear H3K4me3-expression and breast-cancer-specific survival. **c** H3K9ac expression and breast-cancer-specific survival

A high intensity of nuclear H3K4me3 staining (intensity = 3) was found to be correlated with a lower 10-year survival in breast cancer patients (*p* = 0.026; Fig. 5a). Taking into consideration only the patients that died due to breast cancer, we found out, that patients had to have a %Score > 110 to show a significantly better breast cancer-specific survival (*p* = 0.004; Fig. 5b). The cytoplasmic expression of H3K4me3 had no visible effect on the survival of the patients.

The examination of the role of H3K9ac showed no significant effect on the overall survival. Regarding the breast cancer-specific survival, patients with a high %Score had a worse prognosis (*p* = 0.005; Fig. 5c). The threshold needed for significant results was %Score > 190.

### Role of H3K4me3 and H3K9ac on progression-free survival

**Fig. 6:**
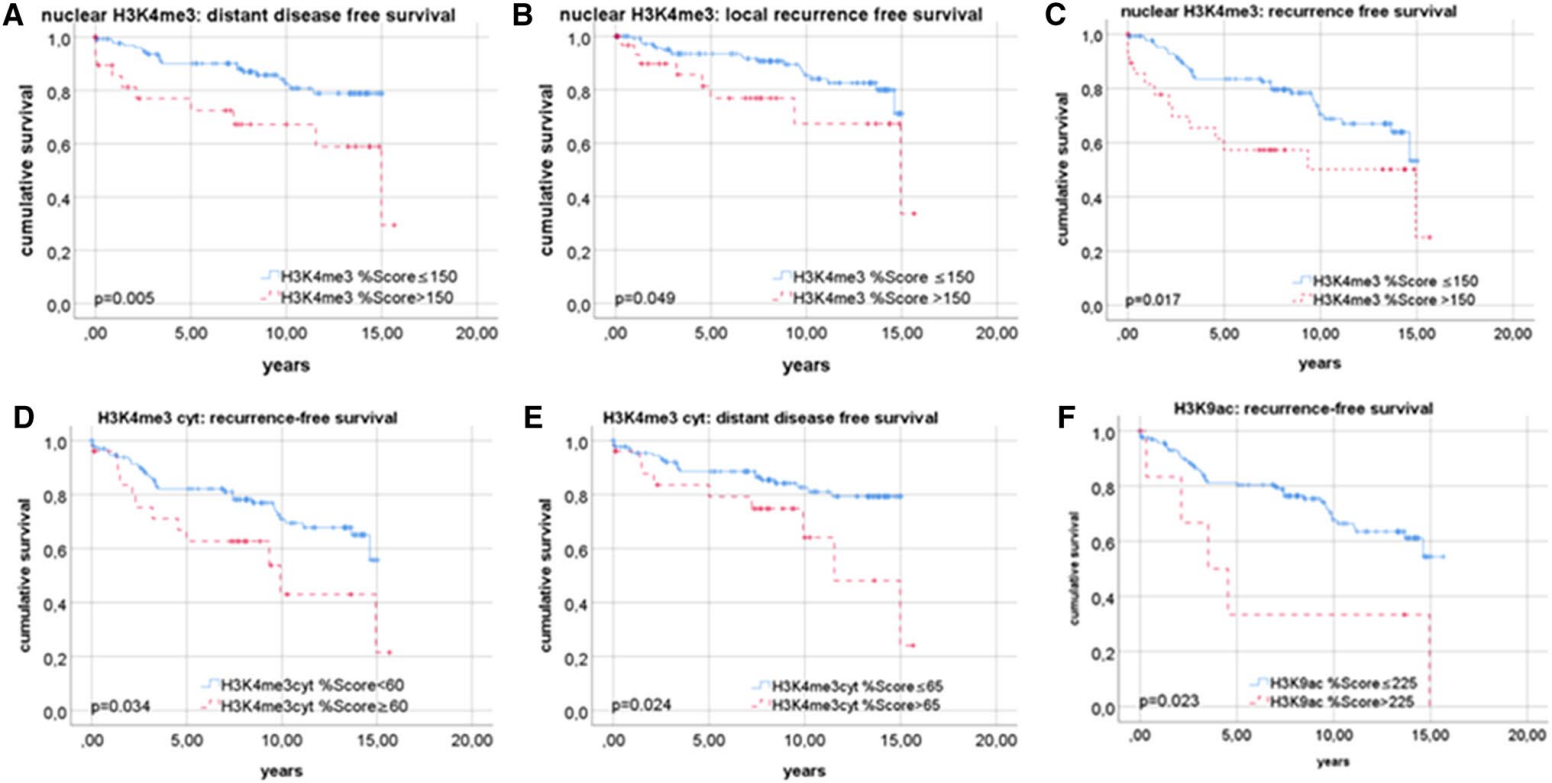
Role of H3K4me3 and H3K9ac for progress-free survival. Nuclear H3K4me3-expression for distant (**a**) and local (**b**) recurrence-free survival. **c** recurrence-free survival in patients with nuclear H3K4me3 expression; recurrence (**d**) and distant disease free (**e**) survival in association to cytoplasmic H3K4me3 expression; **f** recurrence-free survival with H3K9ac expression

In addition to the impact on the patient’s general survival, nuclear H3K4me3 expression was also correlated with the progression-0free survival. The distant disease-free survival, as well as the local disease-free survival, was decreased in patients with %Score > 150 (*p* = 0.005 and p = 0.049; Fig. 6a and b). Combining these two parameters, a significantly shorter general progression-free survival was found in these patients (*p* = 0.017; Fig. 6c).

While the cytoplasmic expression of H3K4me3 seemed to have no impact on the overall survival, it was correlated with a shorter progression-free survival in patients with a %Score ≥ 60 (*p* = 0.034; Fig. 6d). These patients also showed a shorter distant disease-free survival (*p* = 0.024; Fig. 6e). Patients with %Score > 110 also showed a shorter local disease-free survival.

A shorter recurrence-free survival was also found in patients with a high nuclear expression of H3K9ac (%Score > 225, *p* = 0.023; Fig. 6f).

## Discussion

This study showed that specific histone modifications are important in breast cancer patients. H3K4me3 expression was correlated with positive estrogen receptor status, while H3K9ac staining was correlated with positive Her2 receptor status. Although results were highly significant, the correlations themselves were weak.

High H3K4me3 and H3K9ac expression were correlated with shorter breast cancer-specific survival as well as shorter progression-free survival. Overall survival was decreased in patients with high nuclear H3K4me3 staining.

Histone modifications have been of great scientific interest in the past years. After synthesis of histones, posttranslational modifications like methylation or acetylation are performed. These can take place in the nucleus or in the cytoplasm (Annunziato and Hansen 2000; Wu et al. 2012). Posttranslational alterations, which can mainly be found at the loose N-termini, but also within the global domain of histones, have been shown to regulate the structure, accessibility and replication of DNA and play an important role in fundamental cellular mechanisms in the cell cycle (Zhang et al. 2016). Aberrant histone modifications have been linked to the pathogenesis of several diseases, including inflammatory diseases and cancer, as they cause a shift in the gene expression and the overall metabolic state of the cell (Shanmugam et al. 2018).

Previous studies have concentrated on the impact of specific histone modifications on the cell: it has been shown that while histone acetylation is mainly associated with gene activation, methylation can be associated with either repression or activation, depending on its position and level (mono/di/trimethylated) (Kimura 2013; Lee et al. 1993; Vakoc et al. 2006). In this study, we analyzed the impact of H3K4me3 and H3K9ac on the survival of breast cancer patients, as these modifications have been described to be associated with active chromatin (Ruthenburg et al. 2007).

H3K4 methylation is a modification occurring at the fourth lysine residue from the N-terminus of Histone H3. It can be mono-, di- and trimethylated, which adds to the complexity of the analysis of its impact on the genome (Takahashi and Shilatifard 2010). H3K4me3 is generally associated with transcriptional activation and has been proposed as a predictive factor of poor prognosis in several types of cancer, such as liver and cervical cancer (Li et al. 2018). Poorer prognosis has also been described for patients with cervical cancer with a high expression of H3K9ac (Beyer et al. 2017).

By its neutralizing acetyl-group, H3K9ac leads to a de-condensation of the DNA structures and to an activation of transcription (Lee et al. 1993). The effect of an acetylated H3 at position 9 depends on the tumor entity: high H3K9ac levels seem to be associated with a poor prognosis in cervical cancer (Beyer et al. 2017), while patients with glioma have better prognosis with high H3K9ac levels (Liu et al. 2010).

There are much more histone modifications beside H3K4me3 and H3K9ac, which are associated to activation of transcription. For repressive modifications like H3K20me3 and H3K9me3, it was shown, that their levels are elevated in breast cancer cells (Leszinski et al. 2012). Another modification with repressive effect on gene transcription, H3K27me3, Healy et al. could show that it was associated to low grading and inversely to Her-2-neu status (Healey et al. 2014). These results fit to our observance that activating modifications have a positive correlation to the Her2neu status.

In the present study, we showed that H3K4 tri-methylation and H3K4 acetylation are negative prognosticators for breast cancer patients. Even though the mechanisms of histone modifications are not fully understood, several epigenetic therapies have shown great results in cancer treatment. One promising substance class are HDAC inhibitors, which prevent histone deacetylases from detaching the acetyl group from a histone, inducing cell cycle arrest, differentiation and cell death (Eckschlager et al. 2017). The HDAC inhibitors Vorinostat, Romidepsin and Belinostat have been approved by the FDA for the treatment of T cell lymphoma (Zhang et al. 2019). Many studies have concentrated on the combination of epigenetic therapy with well-established therapies and have demonstrated synergistic effects (Cao et al. 2015; Gao et al. 2016; Mann et al. 2007; Marks and Breslow 2007; Rettig et al. 2015). The addition of Vorinostat to Tamoxifen in breast cancer treatment resulted in tumor regression or prolonged disease stabilization in patients who had progressed on prior hormonal therapy (Thomas et al. 2011). In preclinical trials, HDAC inhibitors showed the ability to re-sensitize tamoxifen-resistant cells and prevent hormone therapy resistance (Munster et al. 2011), as well as a potentiation of the immune checkpoint inhibitor blockade in triple negative breast cancer in mice (Terranova-Barberio et al. 2017).

Our results show significant correlations of H3K9ac, H3K4me3 to Her2neu and ER as well as to survival data in breast cancer. As we examined the level of histone modifications at a fixed time by immunohistochemistry, we cannot say, if these results are cause or consequence of the cancer phenotype. Further experiments are, therefore, needed.

Further investigation of histone modifications in breast cancer could lead to a deeper understanding of the molecular mechanisms of cancer development. It could result in reliable screening methods, as well as the identification of new therapeutic targets for breast cancer treatment.

## Conclusions

Histone modifications play an important role in the prognosis of breast cancer. H3K4me3 expression was correlated with positive estrogen receptor status, while H3K9ac staining was correlated with positive Her2 receptor status. High H3K4me3 and H3K9ac expression were correlated with shorter breast cancer-specific survival as well as shorter progression-free survival. Overall survival was decreased in patients with high nuclear H3K4me3 staining.

Several epigenetic therapies have already shown great results in cancer treatment. Further investigation of histone modifications in breast cancer could lead to a deeper understanding of the molecular mechanisms of cancer development. It could result in the identification of new therapeutic targets for breast cancer treatment.

## Data Availability

All data generated or analyzed during this study are included in this published article.

## Abbreviations

H3K4me3: Histone H3 trimethyl K4
H3K4ac: Histone H3 acetyl K9
DCIS: Ductal carcinoma in situ
HDAC: Histone deacetylase inhibitor
LCIS: Lobular carcinoma in situ
OS: Overall survival
DSS: Disease-specific survival
PFS: Recurrence-free survival
Local PFS: Period of time until a local recurrence was diagnosed
DDFS: Period of time until metastasis is diagnosed
